# Impediments to Comprehensive Research on Climate Change and Health

**DOI:** 10.3390/ijerph10116096

**Published:** 2013-11-12

**Authors:** Anthony J. McMichael

**Affiliations:** National Centre for Epidemiology and Population Health, Australian National University, Acton, ACT 0200, Australia; E-Mail: tony.mcmichael@anu.edu.au; Tel.: +61-2-6125-8346

**Keywords:** climate change, health, public health, research, history, modeling, policy, Goldilocks

## Abstract

During every climatic era Life on Earth is constrained by a limited range of climatic conditions, outside which thriving and then surviving becomes difficult. This applies at both planetary and organism (species) levels. Further, many causal influences of climate change on human health entail changes—often disruptive, sometimes irreversible—in complex system functioning. Understanding the diverse health risks from climate change, and their influence pathways, presents a challenge to environmental health researchers whose prior work has been in a more definable, specific and quantitative milieu. Extension of the research agenda and conceptual framework to assess present and future health risks from climate change may be constrained by three factors: (i) lack of historically-informed understanding of population-health sensitivity to climatic changes; (ii) an instinctual ‘epidemiologising’ tendency to choose research topics amenable to conventional epidemiological analysis and risk estimation; and (iii) under-confidence in relation to interdisciplinary collaborative scenario-based modeling of future health risks. These constraints must be recognized and remedied. And environmental researchers must argue for heightened public attention to today’s macro-environmental threats to present and future population health—emphasising the *ecological* dimension of these determinants of long-term health that apply to whole populations and communities, not just to individuals and social groupings.

## 1. Introduction

Astronomers refer to the (presumed) small minority of planets within the universe that lie within the ‘Goldilocks Zone’ around their star. This is the narrow zone—“not too hot, not too cold, but just right”—defined by size of star and distance of planet from it, within which temperatures and conditions are such that surface water can exist on the planet, and hence water-dependent life (*i.e.*, as we know it) is possible [1]. The surface temperature of Venus is 461 °C, while Mars is −46 °C. Earth was around 10 °C warmer than it is today during the dinosaur-dominated Mesozoic, and was manifestly compatible with life. So human-induced global warming, within the currently foreseeable possible range up to around Plus 6–7 °C, is not going to extinguish Life on Earth—unless the warming were to happen so fast that all relocation and biological evolutionary options were precluded.

A similar “Goldilocks” process, at much smaller scale, underlies the way in which climate *change* influences Life on Earth. All living organisms are adapted to thrive within a narrow ‘comfort’ zone of prevailing climatic-environmental conditions. Some can survive temporarily while a little outside that zone; a few will survive when conditions deviate significantly and persistently beyond that comfort zone. (A species with an advanced technological and social culture that could conceivably insulate itself against that external environmental change may be an exception. The only such species known is *Homo sapiens*; but, even so, limits to adaptation will apply).

**Figure 1:**
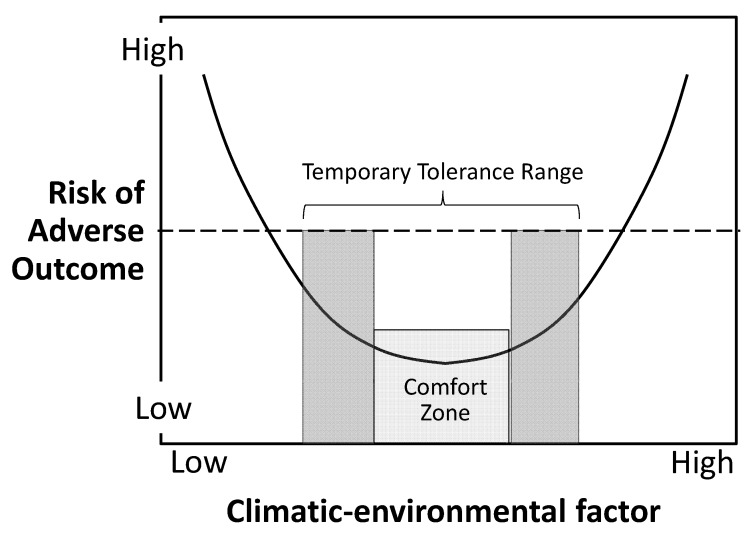
The curvilinear increase in risk of adverse health outcome in relation to exposure excursions beyond and well beyond the organism’s (or ecosytem’s) ‘comfort (Goldilocks) zone’.

The comfort zone phenomenon underlies how climate change influences a wide diversity of human health outcomes. It does so not by adding a linear increment of risk, or by generating or unleashing new risks to health, but by multiplying the preexisting risk or rate of a particular disease by shifting the exposed group above or below its Goldilocks zone. This bimodal phenomenon, shown schematically in Figure 1, is familiar as the typical U-shaped or J-shaped graphs of daily death rates (especially in temperate-zone countries) against daily temperature.

Many other relationships between factors or systems crucial to human population health and climatic conditions display similar U-shaped graphs. These include the photosynthetic productivity of cereal grains and other plants in relation to temperature and rainfall (or soil moisture), the viability of mosquitoes (temperature and humidity), the rate of maturation of various infectious agents within their vector organisms (temperature), and the risk from excesses or deficiencies of freshwater (floods and diarrhoeal diseases, or droughts and dehydration , respectively).

This generic bimodal relationship in the natural world, the source of the life-support system for sustained population health, must be understood by those working in the public health research, policy formulation, implementation and evaluation arenas. We are entering new and largely uncharted waters, requiring responses to health risks that span a greater variety of relationships, complexity and spatio-temporal scale, than our textbooks and professional training have prepared us for. Many of the causal influences of climatic changes or fluctuations on health entail indirect, diffuse or delayed processes, often mediated by the dynamic and interactive complexities of *complex systems* [2]. There will, therefore, be few simple risk-eliminating interventions. Instead, the premium will be on understanding how systemic relationships and processes are likely to vary under various configurations of climate change; where the optimal points of intervention are likely to be; and how best to frame advice for policy-makers to minimize tensions between sectors of government, private enterprise and advisors from various narrowly-focused academic disciplines.

The environmental health agenda has been a consistently high priority for public health during the modern industrial age, stigmatized early on by sanitary crises and Blake’s “dark satanic mills”. Most of the environmental hazards have been of a localized kind, measurable exposures (chemical, radiation or microbiological) acting predominantly via toxicological or infectious insult. In contrast, in this late industrial age humankind is inadvertently testing the limits of human wellbeing, health and indeed viability of regional populations by continuing to deplete and disrupt many of Earth’s biophysical systems [3]. This includes disrupting the climate system by overloading the lower atmosphere with heat-trapping gases that alter its energy-balance function. Despite residual and inherent uncertainties in the science of climate change, and unavoidable limitations in modeling future trends and outcomes, there is now a robust and compelling body of evidence that sustained warming is occurring and that most of it can only be explained by human actions that affect the radiative forcing (“greenhouse”) capacity of the lower atmosphere [4].

In ongoing research to elucidate more fully the health risks from climate change and to improve the prediction of future disease risks and burdens under modeled scenarios of climate change, we are being constrained by three factors. First, we have little historical appreciation of the relative importance of climate-related impacts on the health of human populations over past centuries and millennia. That topic has been little explored and discussed [5]. Second, we are most comfortable, as scientific researchers, with studying things that can be specified and measured, doing so with data from the present and recent past—but not projecting to the future, and when causal relationships are plausible and perhaps even simple and obvious. Third, we have had little experience with modeling how changes in complex relationships and systems are likely to affect future patterns of health and diseases—and yet the future is where the bulk of the adverse health impacts of human-induced climate change will lie.

Each of these constraining factors warrants more detailed consideration.

## 2. Lack of Historical Awareness of Climate-Health Relationships

Natural climate change has been a central influence on the biological and cultural evolution of the human lineage, including, more recently, its sole surviving representative, *Homo sapiens*. Climate change is inscribed in our bones and our minds.

For 3.8 billion years often turbulent changes in the World’s climate largely shaped the evolution of Life on Earth and, during the past 2.5 million years, the evolution of the newly-appeared *Homo* genus. More recently, during the past 11,000 years of the warmer Holocene interglacial period (strictly, epoch), adverse climatic impacts on human population health and survival and on social stability have frequently influenced the fates of societies. Protracted droughts, shifts in regional weather regimes, heavy rains, cold extremes, excessive heat—all have contributed to food shortages, famines, epidemic outbreaks, social disruption, armed conflict and deaths [6].

For example, small temporary shifts in regional temperatures are deemed to have triggered the cascade of ecological events underlying the two great outbreaks of bubonic plague in the Eastern Roman Empire in mid-sixth century CE and in Eurasia in mid-fourteenth century. The typical cascade comprises changes in temperature and rainfall that affect vegetation growth and consequent proliferation and activity of naturally-infected burrowing wild rodents, then transmission of infection to black rat populations that cohabit with humans, the reproduction and longevity of fleas that transmit the bacterium *Yersinia pestis*, and the probabilities of rat-flea-human contacts [7].

Much earlier, during the latter stages of the third millennium BCE, a weakening of the North Atlantic Oscillation—normally the source of moist westerly winds into Europe and the Middle Eastern region—and a shift in the South Asian monsoon coming off the Indian Ocean combined to cause prolonged drying and aridity in Southern Mesopotamia. Food shortages followed, and starvation became widespread. A stone statue to the goddess of epidemics dates from this time. Social disorder and internal conflict increased, and the ancient civilisation of Sumer was then conquered by northern neighbours, the Akkadians [8].

The sustained cooling, of less than 1 °C, during the nadir of Eurasia’s Little Ice Age from around 1570 CE to 1650 CE was associated with widespread extremes of misery, suffering, starvation, epidemic outbreaks, displacement and warfare (in Europe the Thirty Years War, 1618–1648). Historian Geoffrey Parker, focusing on this episode, argues that we can learn much about the influence of climate on human affairs from studying the past. He concludes from his detailed research on the *General Crisis of the Seventeenth Century* that the unusual climatic adversity in Europe during those decades strongly influenced the wellbeing, health and survival of communities and their social and political stability [9]. In China, weather patterns during the 1620s and 1630s were dire: bitter cold, drenching rain that caused waterlogging of fields, and then an extreme drought during 1638–1641. Unrest grew among the starving peasantry and unpaid army, culminating in a rebellion that, in April 1644, gate-crashed the Ming Emperor’s Forbidden City and prompted Emperor Zhengtong to hang himself. Within weeks the Manchurian army was at the gates and the new Ch’ing Dynasty was installed.

This history has not yet been explored fully and systematically, and has therefore attracted little discussion within the ranks of those working on human-induced climate change, its impacts, costs and remediation. Hence we lack a broadly-based awareness of how sensitive many past societies and the health of their populations were to what might appear to us to have been relatively small changes in temperature, rainfall, water supplies and vegetation patterns. However, the recent and rapid expansion in our understanding of the climate system, higher quality and wider-ranging paleo-climatic information, and high-resolution microbial DNA analysis of skeletal samples from ancient epidemic disease victims has opened up new vistas of research and understanding.

The wide-spread lack of awareness of the long history of natural climate change and human health is accompanied by a low level of understanding of the workings of the climate system—something that few of us learnt much about at school. The climate system is complex, dynamic, feedback-rich and made up of many components at different scales. It behoves those doing public health research and policy formulation in this still-unfamiliar topic area to acquire a basic working knowledge of the climate system and the several natural and human-induced influences that affect its capture and retention of heat—that is, its ‘greenhouse’ capacity (knowledge of the climate system should also alleviate the mysteries of the evening television weather reports).

## 3. Epidemiologising (and thus Truncating) the Topic

Current generations of population health researchers and epidemiologists are most comfortable dealing with empirical data, well-defined variables, complexity that reduces to controllable confounding, and intuitively plausible exposure-effect relationships. Much of environmental health thinking, research and practice is predicated on localized exposures to toxic chemical exposures, radiation hazards, and specific microbial contamination. Exposure assessment has become a high-tech specialised activity. Individual gene scanning offers occasional insights (often misinterpreted) about vulnerability differences between individuals.

Much of that body of research methods is far distant from the complexities and tasks of studying and managing risks to health from climate change. As noted above, among the great variety of climate-change risks to health, most entail the multiplication of pre-existing risks. So, if a poor and crowded slum community has a high rate of childhood diarrhea, an increase in temperature and periodic torrential flooding due to climate change will amplify the likelihood of spread of pathogens in water and food. Similarly, if an older-age population in an urban setting has a high prevalence of cardiovascular disease—especially hypertension and heart failure—the impact of a severe heatwave will further amplify the background risk. Typically the risk-function graph turns upwards in exponential fashion.

Conventional epidemiological research methods are readily applicable to the study and estimation of basic risk relationships of this kind, such as between daily temperature and daily death rate or between prior weekly temperature and diarrhoeal disease hospitalisation rates. But many risks to health from climate change are mediated by more complex, sometimes protracted, pathways [2]. A helpful way of thinking about this is with the three categories proposed by Butler and Harley: primary (or direct), secondary (or indirect) and tertiary (or diffuse and delayed), illustrated below [10].

First, though: the point of this section is to caution against choosing ‘lamp-post’ research priorities (referring to the old joke about the drunk who dropped his car-keys somewhere on the footpath, but confined his search to under the lamp-post because “that’s where the light is”). In other words, the research must extend well beyond just those studies that are amenable to conventional epidemiological methods. Otherwise we risk ‘epidemiologising’ the climate-and-health research agenda—as has happened to some extent already with the surfeit of ‘me-too’ studies on daily temperatures and daily health impacts. Instead, for researchers in this field to provide a balanced yield of policy-relevant information requires that they spread their wings and learn about new concepts, methods, the dynamics of genuine interdisciplinary research, and how to handle and communicate unusual levels of uncertainty in ways that can support decision-making.

Examples of research questions relating to secondary ‘indirect’ climatic influences abound. How do we decide whether regional climate change accounts for some or much—or any—of the accompanying ascent of malaria to higher altitudes in tropical mountainous regions? The biological responses of mosquito and pathogen to temperature, humidity and rainfall are very non-linear, and display the above-mentioned Goldilocks bipolar risk; and the background noise from demographic, social and built-environmental confounders is often substantial. Or how do we assign to regional climate change a part of the observed downturn in crop yields in some region, and then transmute that figure into an estimate of climatic influence on under-nutrition and stunted child development? An exploratory example of how such a multi-stage question might be tackled is presented in a recent modeling study by Lloyd and colleagues [11]. First they estimated from the existing literature that, in food-insecure regions, under-nourished children are 60% more likely to die than other children and that growth-stunted children are at a four-fold increased risk of death. They then linked existing estimates, for a medium greenhouse emissions scenario, of the climatic impact on food yield with the above estimates, and projected that the portion of all child deaths from under-nutrition attributable to climate change would double from around 8% to 16% by 2050. Uncertainties in this estimated attributable portion are inevitable since both crop yields and health outcomes are also influenced by many other factors.

Among the third, more diffuse, category of health impacts of climate change, the description, projections and risk estimates will often be of a qualitative kind. As local natural resources dwindle—such as groundwater supplies, river flows, arable coastal land, food yields, safe space for settlements—tensions are likely to build. Although somewhat contentious, considerable evidence indicates that physical conflicts, even overt war, may then break out [12,13]. Displacement of communities will often be associated with such deprivation, social disruption and violence. Hence ‘climate refugees’, such as those displaced into northern Kenya from Somalia during the recent prolonged drought associated with a warmer Indian Ocean surface and weakened monsoon winds [14] will face the many health risks that other refugees and forcibly relocated communities have typically endured [15,16].

## 4. Misgivings about Forecasting Future Health Impacts (Especially for a Plus 4–5 °C World)

Consonant with some of the argument made in Section 2, the medium- to long-term projection of health risks due to an exposure setting that is anticipated to undergo continuing change (*i.e.*, the climatic conditions and associated environmental changes) looms as a formidable task. Such projections are feasible when the exposure experience has ceased, such as past exposure to asbestos, and life-time cohort risks are then calculable. More demanding is to project the future trends in lung cancer rates in China due to the recent and anticipated trend in take-up of smoking, including among women. But, even so, these projections are based on simple causal relationships for which the exposure-effect (or ‘dose-response’) risk equation is well established and not subject to change over time.

The situation in which climate change is the ‘exposure’ is, for most of the health risks of climate change, qualitatively different from those abovementioned examples. However, a minority of the risk relationships are simpler, and amenable to existing epidemiological and statistical research methods. Two obvious examples suggest themselves.

The amplification of some air pollutants (both chemical and organic-aeroallergenic) under conditions of climate change lends itself readily to estimating how future rates of disease and premature death will respond to this climate-influenced change in exposure. Many studies have duly been carried out on this topic, mostly by adapting well-developed methods from countless previous studies of industrial-automotive urban air pollution and health. Forecasting how future changes in patterns of heatwaves will affect daily mortality also has a relatively strong and quantitative empirical base. However, we know little about the shape of the exposure-effect graph at substantially higher levels of cumulative thermal stress in a future hotter world with more extreme weather events, and little about human physiological adaptation over decadal time. Further, with recent uptrends in the prevalence of overweight and obesity, future exposed populations may be more biologically vulnerable to the adverse effects of thermal stress [17].

The public health constituency faces a dilemma here. We must deal with a type of inverse law: the greater the likely health risks that our current climate-disrupting actions are laying in store for future generations more than half a century away in time, the greater the irreducible uncertainty about the type and level of those risks. Today’s policies of mitigation actions and long-term adaptation strategies must therefore be forged under conditions of uncertainty, with limited guidance from estimates of death rates, misery indices, economic costs and so on. Tools to assist decision-making will assume an unfamiliar and discomforting habitus; science will not be able to provide certainty and precision in relation to risk estimates and costings. This dilemma has been well captured by Malcolm Bull [18]:

“*The peculiarity of climate change is that the seemingly natural relationship of policy to time and certainty is inverted: it is precisely because climate change is so uncertain that we have to consider the possibility that it will bring disaster on a global scale, and it is precisely because its impact is long deferred that we must act decisively now.*”

The forecasting of future health risks now faces an added dimension of challenge. As fossil fuel consumption and CO_2_ emissions continue to rise, the likelihood of constraining the average surface warming to not more than 2 °C is fading fast [19,20]. The IPCC’s Fifth Assessment Report (September 2013) concluded that the range of *equally plausible* rises in average global temperature by 2100 includes a 4–5 °C rise [4]. Indeed, on current trends, and given realistic expectations of industrial development trajectories in the next several decades, it now seems likely that average global surface temperature will rise by around 4 °C by 2100 [21,22]. If so, our descendants will face a temperature higher than Earth has experienced for 15–40 million years and a very different landscape. And if collective international efforts, allied with transformative change in ways of living, generating and using energy, do not arrest human-induced climate change, it will continue after 2100. Meanwhile, no matter what, sea-level will certainly continue to rise for many centuries.

So, how might we go about estimating the types, distribution and amounts of health impacts in a Plus 4–5 °C world? The problem is one of complexity, uncertainty and distant time. The further we peer into the future, the more we are beset by uncertainty. How will the world’s climate system *actually* behave beyond the range of historically documented climatic conditions? The climate is not a simple linear system in which the greater the applied force the correspondingly greater the resulting change. Instead, we are in for some climatic-environmental surprises as feedback processes come into play and as various critical thresholds are passed.

The dual task of estimating risks to health and communicating them in the public arena will necessarily take on a very different complexion for these more extreme scenarios. We will need to broaden our frame of risk assessment, work more collaboratively with other disciplines, and rethink the notion of ‘intervention’ within a systems context that provides guidance as to likely optimal points of intervention, but identifies no single definitive levers to pull. The abovementioned paper of Lloyd and colleagues [14] provides an indication of the difficulties of accommodating the concurrent and interactive influences of non-climate variables on future projected food yields and child health. The spectrum of expertise that should be brought to bear on that type of exercise is wide, including agronomists, demographers, nutritionists and others, and their involvement would enhance the integrated inclusion of other putative significant influences on the agricultural and social systems and changes being studied. For epidemiologists and other public health researchers, the vista that is opened up by systems theory and analysis, as a necessary research perspective and method for these increasingly prevalent questions, is both exciting and challenging [23].

## 5. Conclusions

There have been many useful published reviews of climate change and human health. These have imparted a general awareness among environmental researchers in the public health domain that we have an unusually large and complex issue on our hands. That awareness now needs to gain in breadth and sophistication as the climate change problem and its attendant risks continue to increase. The topic of human impacts of climate change is, at last, becoming better recognized and being given more air-play in media and public discussion. This increases the community’s expectations of us, as researchers, to provide well-informed and policy-useful information about present and future health risks. This should encompass information that extends well beyond the rather clichéd frame of current popular understanding about health risks from weather disasters, heatwaves and mosquito-borne infections.

To tackle this task comprehensively and well, the three aspects discussed above must be considered and responded to. That is: understanding better, from history, the range and subtleties of natural climatic influences on the health and social stability of past societies and populations; avoiding the tendency to focusing on the minority of simpler ‘lamp-post’ questions amenable to existing conventional research methods; and developing confidence, skills and commitment to working in a more genuinely interdisciplinary, indeed transdisciplinary, milieu in relation to the long-term forecasting of likely, though uncertain, risks to population health. The opportunity for that more integrative collaboration is strengthening. The long-standing disciplinary divide between the natural and social sciences, with origins in René Descartes’ seventeenth-century prescription that the social and environmental realms should be viewed as separate and unrelated, is receding.

Environmental influences on human health have been inappropriately marginalized in much of the modern public health discourse. We have, at least, moved beyond the late twentieth century’s oft-sterile strategies of ‘health promotion’ that sought to change individual attitudes and behaviours in the face of tidal counter-pressures from the prevailing culture, and we are currently immersed in more strategic thinking geared to the (laudable) ‘social determinants of health’ model [24,25]. Now, environmental researchers in public health must argue for substantial attention to be paid to the escalating and increasingly systemic environmental threats to the present and future health of populations. This advocacy will require fluency with the basic underling science, and an ability to explain the determinants of human health within an *ecological* framework that applies to whole populations and communities, not just to individual, families and social groupings.
